# The Epidemiology and Global Burden of Atopic Dermatitis: A Narrative Review

**DOI:** 10.3390/life11090936

**Published:** 2021-09-09

**Authors:** Hazrina Ab Hadi, Aine Inani Tarmizi, Kamarul Ariffin Khalid, Márió Gajdács, Adeel Aslam, Shazia Jamshed

**Affiliations:** 1 Dermatopharmaceutics Research Group, International Islamic University Malaysia, Bandar Indera Mahkota, Kuantan 25200, Pahang, Malaysia; hazrina@iium.edu.my; 2 Faculty of Pharmacy, International Islamic University Malaysia, Bandar Indera Mahkota, Kuantan 25200, Pahang, Malaysia; aineinani@gmail.com; 3 Faculty of Medicine, International Islamic University Malaysia, Bandar Indera Mahkota, Kuantan 25200, Pahang, Malaysia; k.ariffin@iium.edu.my; 4 Department of Oral Biology and Experimental Dental Research, Faculty of Dentistry, University of Szeged, Tisza Lajos körút 63, 6720 Szeged, Hungary; 5 Institute of Medical Microbiology, Faculty of Medicine, Semmelweis University, Nagyvárad Tér 4, 1089 Budapest, Hungary; 6 Department of Pharmacy Practice, Kulliyyah of Pharmacy, International Islamic University Malaysia, Kuantan 25200, Pahang, Malaysia; adeel.aslam224@gmail.com; 7 Clinical Pharmacy and Practice, Faculty of Pharmacy, Universiti Sultan Zainal Abidin, Besut 22200, Terengganu, Malaysia; pharmacist1992@live.com

**Keywords:** atopic dermatitis, atopic eczema, prevalence, incidence, epidemiology, risk factors, filaggrin, immune dysregulation

## Abstract

The global epidemiology of atopic dermatitis (AD) in the current decade (2009–2019) has not been extensively reported. Epidemiological studies play an important role in presenting the risk factors of AD, as detailed prevalence and incidence data could demonstrate the burden of disease in the population of adults, adolescents, and children in different geographical regions. Thus, the primary objective of this review was to assess and summarize the epidemiological studies of the prevalence and incidence of AD in different age groups, focusing on data from studies published for 2009 to 2019. After a thorough literature search, six countries were identified from African, Asian, and European regions respectively, who published studies on AD. In contrast, only two studies were identified from Australia and New Zealand, three countries from North America and two from South America published AD studies, respectively. The highest prevalence of AD from included studies was noted among Swedish children with 34%, while the lowest prevalence was in Tunisian children with 0.65%; studies reporting incidence data were far less numerous. A common trend in the prevalence of AD was that children would have a higher prevalence as compared to adolescents and adults. The severity and morbidity of the disease showed variance with age, sex, socioeconomic characteristics, geographical location, and ethnicity. Environmental factors played an important role as causative agents in AD. The risk factors that were proven to cause and induce AD were skin barrier impairments due to FLG mutation, changes in the environment, and diet. FLG mutation may impair the skin barrier function by disruption of pH and hydration maintenance of the skin. Lastly, there were only a few studies on the incidence of AD in the 21st century. Therefore, epidemiological studies on childhood and adulthood AD in different continents are still needed, especially on the incidence of AD during adulthood.

## 1. Introduction

Atopic dermatitis (AD), a condition which also goes by the name ‘*atopic eczema*’, is a chronic, inflammatory, and eczematous skin disease. The term ‘*eczema*’ is related to the eczematous nature of this disease, which presents with rusting, serous oozing, and blister formation, in addition to erythema and scaling [1]. AD is commonly diagnosed in children but it may also affect patients in their adult years. It has been reported that children diagnosed with AD may also present with asthma and allergic rhinitis, which are the common comorbidities of AD in infancy and/or in early childhood. In few instances, the development of AD is seen as an initiation mechanism, and thus paves the way for bronchial asthma and allergic rhinitis, collectively called ‘Atopic March’ [2].

### 1.1. Pathogenesis of AD

It has already been established that the skin is the biggest organ of our body, and it acts as physical protective barrier from external harm. A combination of functional defects in skin barriers, along with genetic predisposition and immune dysregulation seems to be the causative factors for the emergence of AD [3]. The skin comprises of three layers, namely the epidermis, dermis, and subcutaneous tissue. The epidermal layer may be further divided into five sublayers, including the stratum corneum (SC), stratum lucidum (SL; only found in the epidermis of the soles and palms), stratum granulosum (SG), stratum spinosum (SS), and the stratum basale (SB) [4]. The mentioned ‘barrier’ role of the skin is predominantly associated with the role of SC in the epidermal layer of the skin. The SC has two main functions, including the prevention of excessive trans-epidermal water loss from the outer layer of the skin and being the first-line of defense from pathogenic compounds and exogenous chemicals that could induce an immune reaction when they permeate into the epidermal and dermal layer [5].

**Figure 1:**
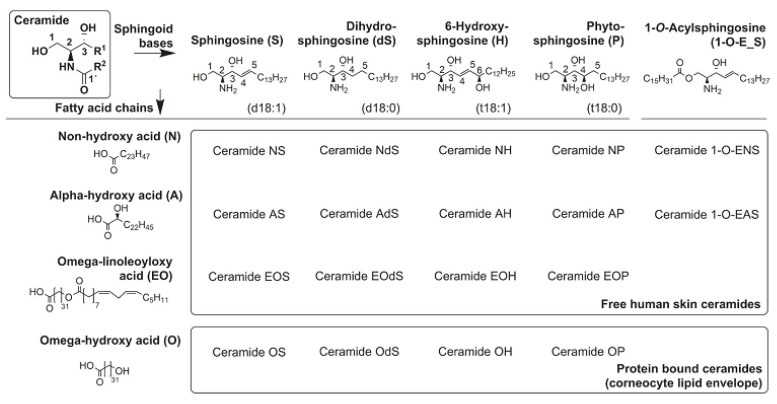
Chemical structures of the main components of the stratum corneum (SC) ceramides and their shorthand nomenclature.

**Figure 2:**
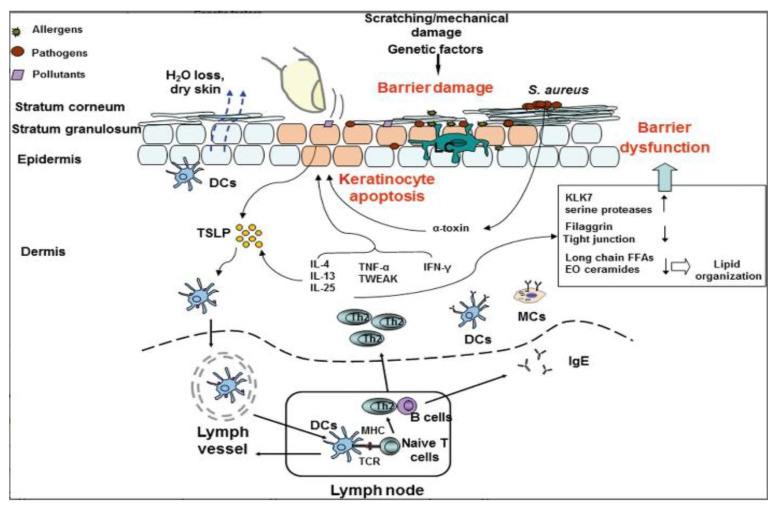
Graphic summary of effects of skin barrier on the pathogenesis of atopic dermatitis (AD). Genetic and immunologic as well as mechanical factors such as scratching induce skin barrier damage, allowing contact of skin resident antigen-presenting cells to allergens, bacterial, and viral antigens as well as other environmental factors. Activated antigen-presenting cells migrate to lymph nodes and prime naive T cells into Th2 cells. Elevated Th2 cytokines, together with TNF-α and IFN-γ further damage skin barrier functions by inducing apoptosis of keratinocytes as well as impair the function of tight junctions and promote Th2 responses by enhancing TSLP expression of epithelial cells. Moreover, colonizing pathogens, such as *Staphylococcus aureus*, impair barrier function through the release of virulence factors to induce keratinocyte death and to boost Th2-type inflammation. Together, genetic and immunological factors contribute to the skin barrier dysfunction and play a major role in the pathogenesis of AD.

The SC has a specific lipid composition (which consists of ceramides (CERs), cholesterol (CHOL), and free fatty acids (FFAs), and a histological organization, which—if disturbed—may lead to impaired skin barrier function [6]. Impaired SC barrier function may result in several skin disorders, such as AD. Ceramides keep skin hydrated, they help to soften the upper layer of the skin and they also support the skin barrier function and may be crucial in preventing inflammation in the skin. While ceramides also boost healthy skin, they are also crucial in repairing damaged/diseased skin [5,7]. The deviation of lipid composition in AD skin from the composition of healthy skin will eventually lead to haywire in the organization of lipids. CERs may be characterized by their sphingoid base and their acyl chain moieties. The sphingoid base may either be sphingosine (S), phytosphingosine (P), 6-hydroxy-sphingosine base (H), or dihydrosphingosine (DS), while the acyl chain may either be non-hydroxy fatty acid (N), an α-hydroxy fatty acid (A), or an esterified ω-hydroxy fatty acid (EO) [5,7]. The various combination of these two components may lead to 12 subclasses of CERs. The most abundant subclasses reported in the healthy skin are the sphingoid base of phytosphingosine (P) coupled with an acyl chain of non-hydroxy fatty acid (CER-NP) (~25–30 molar%). The very long ceramide moiety, coupled with esterified ω-hydroxy fatty acid (CER-EO/acyl-CER)—which are seen in the subclasses EOS, EOP, EOH, and EODS—are known to be essential for appropriate SC barrier function [5,7,8]. The chemical structures of the main components of the SC ceramides and their shorthand nomenclature are presented in Figure 1 [9]. Lipid organization of the SC is another relevant factor, which is important in maintaining the skin’s function as a barrier. There are two types of lipid organizations that play a role, namely the lamellar and lateral organization. The lamellar phases are present in the SC in a broad–narrow–broad fashion, which may extend as long as ~12 nm. The lamellar phase comprises of two phases which are the long periodicity phase (LPP) and the short periodicity phase (SPP). The short periodicity phase (SPP) is characterized by a with 5–6 nm, while the long periodicity phase (LPP) is characterized by a 12–14 nm lamellar repeat distance, respectively [5,6,7,8,9]. van Smeden and Bouwstra reported that acyl-CERs are crucial for LPP formation. This indicates the critical relationship between SC lipid organization and SC lipid composition. The lateral organization is also important in maintaining the structure of the SC [5]. SC lipids exhibit a lateral organization with three possible morphologies, including liquid (not in order), hexagonal (less dense order), or orthorhombic (very densely packed). The lateral organization is the arrangement of the lipids within the plane of the lamellae and perpendicular to the lamellar lipid organization (Figure 2) [5,6,7,8,9,10].

The differences that may be observed in AD skin—when compared with a healthy skin—are associated with their lipid composition and organization [5,6,7,8,9,10]. In AD skin, there is an increase in CER subclasses AS, AH, AP, ADS, NS, a decrease in CER subclasses NP, NH, acyl-CERs (CER-EO), an increase in short-chain CERs, and a decrease in long-chain CERs. For fatty acids, there are more short-chain FFAs, fewer long-chain FFAs, an increase in mono-unsaturated fatty acids (MUFAs), and a decrease in hydroxy-FFAs. The lateral organization of SC demonstrates fewer lipids with orthorhombic packing, more lipids having a hexagonal lipid packing, and less conformational clustering of lipids. Furthermore, AD skin has a reduced repeat distance of lamellar phases [5,6,7,8,9,10,11].

Filaggrin (FLG) or ‘filament aggregating protein’ is a protein within the SC, which has an important in maintaining the structural integrity of the SC. The interior of the corneocytes consists mainly of keratin filaments aggregated by the FLG protein [12]. Alongside this protein, there are also other proteins important in maintaining the skin barrier, including filaggrin, corneodesmosin, desmoglein-1, desmocollin-1, and transglutaminase-3. FLG is important for the structural and mechanical integrity of the SC. Moreover, the degradation products of FLG are also essential and play a role in maintaining the skin barrier. They are involved in the maintenance of skin hydration and the acidic pH of the skin, both important for the optimal activity of enzymes involved in the reduction of skin inflammation, lipid synthesis, and shedding/peeling of the outer layer of the skin [12,13]. The deficiency of filaggrin has been described to essentially contribute to the pathogenesis of atopic dermatitis (AD), one of the most frequent chronic eczematous skin diseases in childhood and adulthood [14]. The causes of FLG deficiency are multifactorial: they are either caused by a genetic mutation (primary) at the epidermal-differentiation complex (EDC) on chromosome 1q21, or other secondary factors, which are influenced by the external environment, including low humidity or mechanical damage. AD skin—which is deficient in FLG—has an increased SC permeability, due to the disturbed intracellular matrix of corneocytes, which subsequently leads to impaired SC function [15]. As it is well-known, the immune system as a whole may further be divided into innate immunity and adaptive immunity. When there is a defect in innate immunity, the skin is more susceptible to be re-infected for the predisposed AD patients, as their body is unable to recognize pathogens [16]. In AD skin, there is an impairment of pattern recognition receptors (PRRs), such as Toll-like receptors (TLRs) and nucleotide-binding oligomerization domain-like (NOD-like) receptors. Downregulation of these two receptors will impair the production of transcription factors that promote gene expression of pro-inflammatory proteins, such as cytokines that are necessary for protection against pathogens [17,18]. In adaptive immunity, this is a far more complicated process. T cells, especially CD4^+^ T-helper cells, play an important role in the continuation of the AD response, which are mediated by TH_2_, TH_22_, and TH_17_ cells as the primary drivers of acute AD, while TH_1_, TH_2_, and TH_22_ cells drive the chronic phase of the disease. TH_17_ cells are the source of Interleukin-17 (IL-17), a cytokine that is known to be relevant in the pathogenesis of psoriasis has been recently identified in acute and chronic AD lesions. Increased IL-17 and IL-22 expression are usually associated with acute and chronic AD lesions. A higher IL-22 expression is commonly found in acute lesions. Pediatric patients show a higher expression of TH17 and TH9 cytokines than adult patients with AD. IL-5 levels were also found to also be significantly higher in the serum, as well as skin lesions of patients with AD [17,18]. In a recently reported review by Dubin et al., IL-4, IL-13, and IL-31 inflammatory pathways are also implicated in immune and barrier defects [19].

### 1.2. Clinical Manifestations of AD

The name ‘eczema’ gives a general idea on the way this disease presents itself in patients. Atopic eczema or AD is a pruritus and eczematous condition of the skin. Relapsing dry skin, and severe pruritus are some of the most important hallmarks of the disease. However, the clinical presentation is highly variable, depending upon the patient’s age and severity of illness. Acute lesions are characterized by pruritic papules with erythema, peeling of the skin, and serous exudation. Skin thickening, leathery modification of the affected areas from chronic scratching (lichenification), and small linear-like cleaving of skin may develop over time [20,21]. In infants and young children, the disease typically presents with pruritic, red, scaly, and crusted lesions on the extensor surfaces, cheeks, or scalp. Common triggers include various environmental stimuli, including heat, sweating, anxiety, frustration, and infections. Although rarely, food allergy may also be a trigger; however, this factor is commonly over-diagnosed among children [22]. In older children and adolescents (2 to 16 years), AD is characterized by less exudation and often demonstrates as lichenified plaques in antecubital and popliteal flexures and neck. The sides of the neck may show a reticulate pigmentation, the so-called ‘atopic dirty neck’. In adults, atopic dermatitis is considerably more localized and lichenified. The areas involved are the skin flexures in most cases. Less frequently, the dermatitis may also involve the face, neck, or hands [23]. To sum up, impaired SC, genetic mutations, and dysregulation of the immune system are the three main causative factors of AD. The clinical manifestation of AD usually varies between adults and children, but some common clinical features, like the relapse of dry and severe pruritus of the skin, may be identified. The incidence and prevalence of AD show significant variance among children and adults, usually showing that children have higher prevalence compared to adults. Herein, prevalence means the number of cases of a disease in a specific population at a particular time point or over a specified period of time. On the other hand, incidence is the rate of new cases of a disease occurring in a specific population over a particular period of time. Moreover, different socio-demographic factors among AD patients may also influence the incidence rate of AD [20,21,22,23].

## 2. Risk Factors for Atopic Dermatitis (AD)

### 2.1. FLG Protein Deficiency

A protein important in maintaining pH and moisture in the skin is filaggrin (FLG), which is situated within the SC. The degradation products of FLG are also essential, and play a pivotal role in maintaining the skin’s barrier function. The mechanical and structural integrity of SC is also upheld by the FLG protein. AD skin—which is deficient of FLG—has an increased SC permeability, due to the disturbed intracellular matrix of corneocytes which then leads to impaired SC function [12]. There were two studies reporting that FLG deficiency was associated with an early onset of AD [24]. When a mutation affecting FLG protein occurs, it may lead to insufficiency in the production of FLG, thus impairing its function in maintaining the skin barrier function. Various studies were conducted to observe the prevalence and mutation of the FLG as a causative of AD. A study performed among Japanese and Korean AD patients reported that 27% and 21.1% in respective patients carrying various numbers of FLG mutations, ranging between zero to nine different types of FLG protein mutations [25]. Another study performed in Poland reported that in 30% of AD patients, 22 different mutations were detected. Similarly, a study performed by Rupnik et al. stated that 11% of AD patients in Slovenia carried at least one FLG mutation. A high rate of mutations of the FLG gene in patients with clinically diagnosed AD was observed in their studied population [24].

### 2.2. Climate

Various studies performed in different parts of the world are important to observe the incidence and prevalence of AD in different climatic regions, to ascertain whether the tropical climate with hot and humid air like in the Southeast region of Asia or dry and cold in Northern America has a significant effect on AD epidemiology. Based on the National Survey of Children’s Health and climate variables in the USA, it was reported that the prevalence of eczema was significantly lower in areas with higher UV indices, humidity levels and mean temperatures, and less indoor heating and precipitation [26,27]. Moreover, a study done on the Ogasawara Islands, which is located 1000 km south of mainland Japan, had a lower AD prevalence among children when compared to previous reports from mainland Japan [28]. It was proposed that the reason for this difference was influenced by the warm and humid climate associated with Ogasawara Island. Other than that, the children were exposed to more UV rays due to more activities performed at the beach. UV rays are actually one of the effective therapy in treating severe AD. Therefore, the constant UV exposure may contribute to the lesser prevalence of AD among Ogasawara children. It was also reported that the onset of AD among Norwegian children was primarily observed during the winter and spring seasons [29]. Various seasons have a different role and effect in causing AD. The winter season has a very low temperature, hence, AD could be caused by the drying of the skin due to these temperature stimuli. The spring season is commonly associated with pollens, which is one of the main group of allergens that could cause and induce AD.

### 2.3. Air Pollution

A plethora of studies were aimed to compare the prevalence of AD between urban and rural areas in a certain country. These studies are beneficial in associating the prevalence of AD to the risk factor of air pollution. Studies done in Japan, Korea, China, Taiwan, Germany, and France concluded that populations in urban areas had a higher prevalence of AD compared to those living in rural areas [28,30,31,32,33,34,35]. A study concluded that AD was significantly and positively associated with persistent exposure to benzene, nitrogen dioxide (NO_2_), nitrogen oxides (NO_x_), and carbon monoxide (CO) among French children [30]. Other than that, a study conducted by Xu et al. stated that Shanghai children that live in core urban areas (which was in the Xuhui Tianping region) had the highest prevalence of AD of 10.2%. There is a significant decrease in prevalence when compared to children who lived further away from the urban areas (which was in the Chongmin Baozhen region) with a prevalence of 4.6% [31]. As previously stated, Ogasawara Island children had a lower AD prevalence than the children who lived in mainland Japan due to the fact that Ogasawara Island is considered as a rural area [28]. A study reported that there were significant associations between exposure to air pollutants and the incidence of eczema symptoms among elderly German women after the age of 55 [36]. Additionally, a study in Taiwan concluded that there was a modest association between sulfur dioxide (SO_2_), nitrogen dioxide (NO_2_), ozone (O_3_), and carbon monoxide (CO) exposure with the development of AD among the adult Taiwanese population [34]. Lastly, Yi et al. reported that AD among Korean children in Seoul could be caused by the traffic-related air pollution TRAP; in their study, the prevalence of AD was 15.95% [35]. It has been proposed that the role of air pollutants in causing AD could target both the fetus during pregnancy, infants, and children. After birth, the fetus will produce reactive oxygen species (ROS) when in contact with air pollutants, thus inducing oxidative stress in the skin, which leads to damage in proteins and lipids in the epidermis. Consequently, it may cause impaired skin barrier function. However, this hypothesis has not been backed up by epidemiological or experimental evidence so far [37].

### 2.4. Food Allergies

One of the pathogenetic mechanisms of AD is immune system dysregulation. The risk factors of food allergies have a close relationship with this pathogenesis. The sensitization occurs mainly at the level of the gastrointestinal wall, and is related to the dysfunction of adaptive immunity. The exacerbation of AD by food components occurs when the immune responses were triggered by increased binding of antigens to the immature gut microvilli, thus leading to increased intestinal permeability of antigens into the body [38]. The microflora in the intestinal tract will act as a strong antigen and exacerbate AD during the consumption of the particular foods and nutrients; hence, causing inflammation [39]. Foods have been shown to be a trigger in 20–30% of cases of moderate-severe AD. The most common food allergens identified were cow’s milk, wheat, soy, cod, egg, and peanut [40,41]. Two studies conducted among Iranian children and the Southern population of Thailand concluded that the most common food allergen in AD patients were egg whites and cow’s milk [42,43]. The occurrence of egg white (35.7%) and cow’s milk (26.8%) as an allergen among Iran children were similarly high [42]. While in Southern Thailand, children AD patients aged 2 months to 5 years had a prevalence of total food sensitization of 60%, of which the highest two were egg white (56.8%) and cow’s milk (40%) [43]. Similarly, a study in Taiwan concluded that sensitization to a food allergen—especially egg white—showed a statistically significant risk in association with eczema [44].

### 2.5. Obesity

The correlation between obesity and AD is still controversial, according to the currently available literature, showing mixed results. Further mechanistic studies are needed to clarify the relationship between these two variables. The reasons behind this relationship are still debatable; however, there is a study that hypothesized that increasing in body weight leads to higher levels of circulating interleukin-6 (IL-6), leptin, and tumor necrosis factor alfa (TNF-α) [45]. This is due to the fact that obese patients will have a higher amount of white adipose tissue than their non-obese counterparts, thus, they will produce more adipokines, as they (leptin and adiponectin, IL-6 and TNF-α) are actually secreted by white adipose tissue. Hence, the increased production of IL-6 and leptin will downregulate the activity of regulatory T–lymphocytes (T_reg_ cells), which will subsequently lead to reduced immunological tolerance to antigens [46,47]. A few studies were published in observing the correlation between obesity and AD. Firstly, it was reported by Lin et al., that Taiwanese adolescents with high BMIs (which were classified as higher than 23 kg/m^2^) were more prone to develop AD than their friends with normal BMI measurements (classified as between 17 and 21 kg/m^2^) [48]. However, the differences were not statistically significant. Next, obesity was significantly associated with AD in young Korean adult women in the age range of 19 to 40 years old but the authors were unable to find the relationship between obesity and AD in men [32,33]. There are also studies that focused on children: a Spanish study has reported a positive association between AD and obesity among six to seven-year-old schoolchildren [49].

## 3. Global Epidemiology of AD

### 3.1. Background

In the past decades, reports on the incidence and prevalence of AD had mainly included surveys of the general population, or adult-specific and/or children-specific studies. Only 25% of children diagnosed with AD will continue to be affected by the disease in adulthood, either as a continuous illness presentation or by having a relapse of symptoms after some symptom-free years. Approximately 75% of patients with childhood-onset AD will have their condition spontaneous cease before reaching adolescence [50]. The epidemiological trends of AD among children and adults vary. Two recent studies conducted in Germany presented a lower prevalence of AD among adults compared to children: prevalence of AD among children in Germany was 10.35% while among adults is 3.67% [51,52]. The incidence and prevalence of AD among children changes across the different parts of the globe, based on a comprehensive report containing data corresponding to 12 years, namely Phase 1 (1992–1997) and Phase 3 (1999–2004) [53]. These studies had played an important role in clarifying the epidemiological aspects of AD, such as highlighting the most important risk factors. According to Noordzij et al., prevalence studies are important in demonstrating the burden of disease (BoD) in a certain population in terms of morbidity, life expectancy, and quality of life (QoL). This report has also concluded that severity and morbidity of the disease also varies with age, sex, socio-economic characteristics, geographical location, and ethnicity [54].

For incidence data, a research performed in Denmark and Sweden reported that the incidence rate of AD is stable among Swedish and Danish children [55]. Furthermore, another comprehensive study on the incidence rate of AD in the United Kingdom (UK) also reported a stable incidence rate among children between 1997 and 2015 [55]. However, the incidence rate showed pronounced differences based on the various sociodemographic factors during the span of the 18-year study period. For example, in infants younger than one year of age, boys showed a higher incidence rate of 30%, when compared to girls [56]. In addition to exploring the risk factors associated with this disease, the objective of our present paper was to summarize the reported prevalence and incidence of AD among adults, adolescents, and children in the world.

### 3.2. Literature Search Results

Based on the studies included from the literature, the prevalence of AD has shown pronounced variance across the globe. The prevalence of AD between 2009 and 2019 was reported using a variety of methods in measuring the prevalence of AD; for example, the International Study of Asthma and Allergies in Childhood (ISAAC) questionnaire, Hanifin and Rajka’s diagnostic criteria, the UK Working Party’s diagnostic criteria, individual physician-diagnosed criteria and also parent reports [57]. However, reports on the incidence of AD were far less common, with data available only from Norway, Denmark, Sweden, China, and Germany.

Furthermore, many risk factors reported for AD in these studies had a close relationship with the three main pathogenetic factors of AD, which were impaired skin barrier function, genetic mutations, and immune dysregulation. The skin barrier dysfunctions were associated with the FLG mutation which caused a deficiency in FLG protein synthesis and thus, impaired the role of FLG protein in maintaining pH and the hydration level of the skin. Environmental factors also played an important role as a causative agent in AD. Many cases reported that air pollution and local climates had an impact on the prevalence of AD. Lastly, immune dysregulation reactions were reported, associated with food allergies, and obesity.

### 3.3. Prevalence of Atopic Dermatitis (AD)

According to the United Nations (UN), Department of Economic and Social Affairs, Statistics Division, the World may be divided into five major geographical areas, which are Africa, America, Asia, Europe, and Oceania. Each of these areas have their own geographical subdivisions as such:

1. Africa: Six subdivisions (Northern Africa, Sub-Saharan Africa, Eastern Africa, Middle Africa, Southern Africa, and Western Africa)
2. America: Five subdivisions (Northern America, Latin America, and the Caribbean, Caribbean, Central America, and South America)
3. Asia: Five subdivisions (Central Asia, Eastern Asia, South-eastern Asia, and Western Asia
4. Europe: Four subdivisions (Eastern Europe, Northern Europe, Southern Europe, and Western Europe)
5. Oceania: Four subdivisions (Australia and New Zealand, Melanesia, Micronesia, and Polynesia)

Based on the available studies, only six African countries—i.e., Tunisia, Rwanda, Namibia, Gabon, Ghana, and Senegal—reported on the prevalence of AD. From the American region, Latin America, the United States of America (USA), Canada, and Brazil highlighted the prevalence of AD. From the Asia region, six countries published studies regarding the prevalence of AD which were China, South Korea, Japan, Malaysia, Taiwan, and India. Moreover, seven countries from Europe also contributed to this literature search which were the United Kingdom, Denmark, Sweden, Poland, Germany, and France. Finally, Australia and New Zealand were the countries to represent prevalence data from Oceania. The prevalence of the studies were considered as point prevalence during analyses unless stated otherwise.

### 3.4. Africa

**Figure 3:**
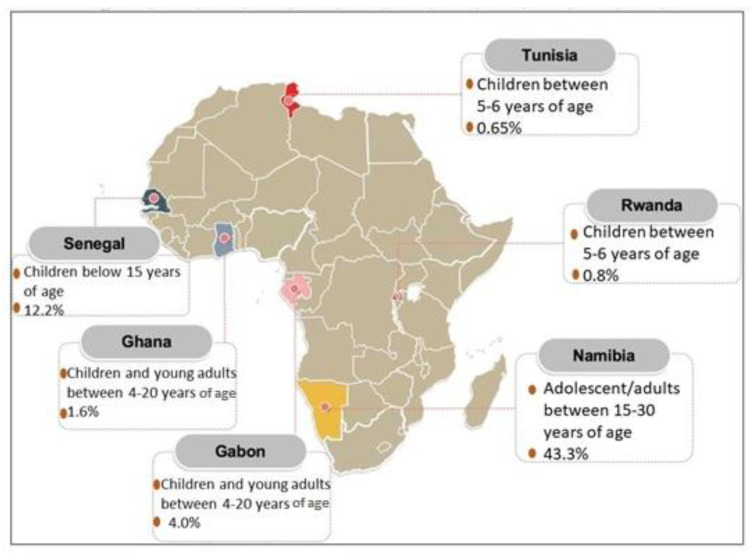
Prevalence of AD in Africa based on the reports published between 2009 and 2019.

Figure 3 summarizes the available prevalence data on AD from the African region. A study was conducted in Ghana, Gabon, and Rwanda during the year 2007 to assess the point prevalence of AD among children: a total of 4839 schoolchildren between the ages 4 and 20 years of age were observed. They have found a point prevalence of 1.6%, 4.0%, and 0.8% respectively [58]. In Tunisia, 1617 children were involved in determining the prevalence of AD in 2007. A result of 0.65% prevalence of AD was reported among five to six years old Tunisian children [59]. Next, Namibian adolescents aged between 15 to 30 years old reported having a prevalence of 43.3% during a study conducted in 2014 [60]. This prevalence value was the highest reported on this continent. Lastly, a study among 321 Senegalese children with ages under 15 years old presented with a prevalence of 12.2% [61]. The lowest prevalence on this continent was seen in children from Rwanda. Based on the abovementioned reports, the average prevalence for African children would be 3.85%, the majority of the prevalence reported are amongst the children population. Unfortunately, there were no available studies that reported on the adult population from African countries.

### 3.5. America

In the USA, many prevalence studies were conducted among both children and adults. In the adult population, three AD prevalence studies were published during the years 2013, 2015, and 2019. Their respective prevalence rates were reported as 10.2%, 7.2%, and 7.3%, respectively. It could be said that the prevalence of AD among adults decreased from 2013 to 2015. However, there was a slight increment in 2019. All three of these studies had a study population of patients older than 18-years-old [26,27,62,63]. Children in the USA who were the age of 5, 9, and 15 years old were reported to have a prevalence of AD at 15%, 15.1%, and 14.5%, respectively [64]. Therefore, the average of these prevalence data may be summarized as 14.8%. The prevalence of children from zero to five years old is significantly higher which was reported at 24% [65]. This trend is reasonable, according to previous studies and studies that will be discussed later which described younger children had higher prevalence compared to older children, adolescents, and adults.

In Brazil, there were two studies reporting on the prevalence of AD in children and adolescents. A study conducted on 3600 children between the ages of six and seven years old reported a period prevalence of 9.6% in the last 12 months [66]. They also reported a prevalence of 11.4% when AD was diagnosed by a licensed physician. Toledo et al. reported that the prevalence of AD among 13- to 14-year-old adolescents in São Paulo has been reduced from 2005 to 2012, corresponding to prevalence rates of 16.2% to 3.4%. The study participants were represented by 1039 adolescents from São Paulo, Brazil [67]. There are also two other studies conducted in the Latin America region, which reported on the prevalence among infants, children, and adolescents. Infants of 12- to 15-months old gave the highest prevalence in this region which was 18.2% [68]. Children aged between 6–7 years in Latin America had an AD prevalence of 11.3%, while adolescents aged between 13–14 years had a lower prevalence of 10.6% [69].

**Figure 4:**
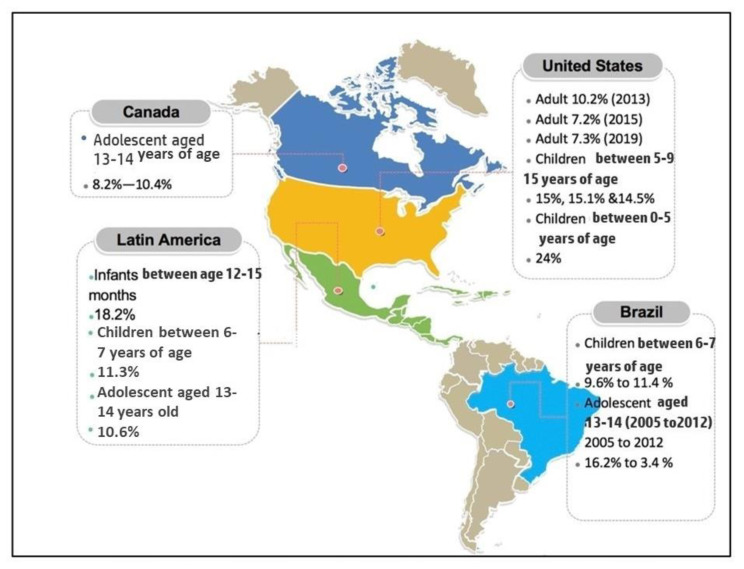
Prevalence of AD in America based on the reports published between 2009 and 2019.

A study performed by Wang et al. reported the prevalence of AD among 13- to 14-year-old adolescents in five cities in Canada (which were Vancouver, Saskatoon, Winnipeg, Hamilton, and Halifax) [70]. This study comprised of the data corresponding to 8334 adolescents and was conducted in February and December of 2008. The prevalence rates were in the range of 8.2–10.4%. The most prevalent cities for AD were Winnipeg and Vancouver. Figure 4 summarizes AD prevalence data corresponding to the American region.

### 3.6. Asia

**Figure 5:**
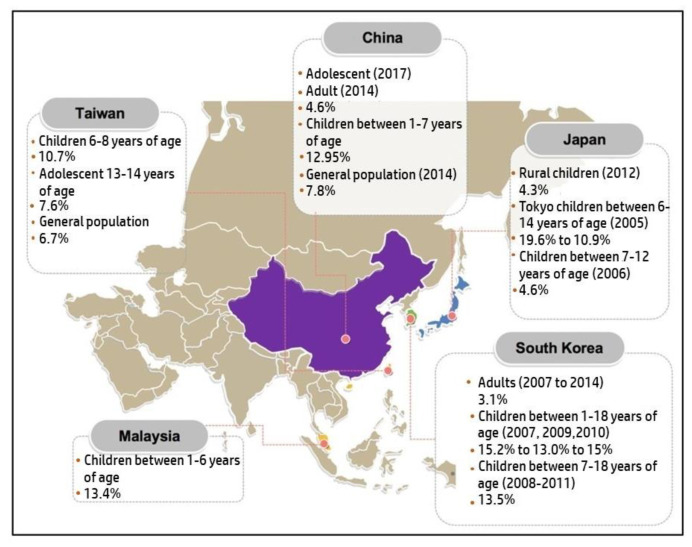
Prevalence of AD in Asia based on the reports published between 2009 and 2019.

Multiple studies were published from Asian countries, reporting on the prevalence of AD in their respective regions, with their results summarized in Figure 5. There were a total of five countries in Asia, which reported the prevalence of AD among children, adolescents, and adults. The countries that contributed for data in Asia were South Korea, Malaysia, Taiwan, China, and Japan. In South Korea, a study done by Lee et al. measured the prevalence of AD in 2116 children, 1–18 years of age, during 2007, 2009, and 2010 [71]. There was a decrease in prevalence from 2007 (15.2%) to 2009 (13%) but followed by a subsequent increase to 15% in 2010. Other than that, another study reported a similar result among children one to 18 years old in Korea, which was 13.5% [72]. Adolescents in Korea reported to have AD prevalence of 23.1%, which was the highest reported prevalence value in Asia. A total of 75,643 adolescents with the age range of 13- to 18-years-old were studied in their study [73]. Moreover, in adults, Lee et al. published their results on 47,350 adults from 2007 to 2014, which have found that adult Koreans had an AD prevalence of 3.1%; this prevalence was the lowest reported in the Asia region. There is only one study reporting on AD prevalence in Malaysia in the recent years. The study was conducted on 384 children, aged between one to six years old and reported a prevalence of 13.4%. The prevalence of AD in Malaysian children was similar to the Korean population; nevertheless, it was one of the highest prevalence values in Asia. The study also reported that Malaysian Chinese were the dominant participants; however, Malays were discovered to have the highest prevalence among Malaysian children [74]. In Taiwan, 24,999 Taiwanese children between 6–8-years of age were studied. The reported AD period prevalence within the time period of 2007 to 2008 was 10.7% [75]. Lin et al. conducted a study among 74,688 junior high school students within the age range of 13 to 15 years old, which gave a prevalence of 7.6% [48]. Lastly, the general population of Taiwan reported to have an eight-year period prevalence of AD, which was 6.7% during a study conducted from 2000 to 2007. The total participants were 997,729 Taiwan citizens [76]. In China, the most recent study was conducted among 8226 adolescent students in September 2017. Xiao et al. stated that the prevalence was 2.5% [77]. Following that, a study done among Chinese 8758 outpatient adults stated that the outpatient overall AD prevalence was 4.6%. Chinese children between the age of one to seven years old had an AD prevalence of 12.94% reported by a study done on 13,998 Chinese children in 2014 [78]. Lastly for China, the general population had an AD prevalence of 7.8% which the result was reported from a study done by Wang et al. from 1 to 30 September 2014 [79].

In Japan, the rural children who lived in Ogasawara Island gave the lowest AD prevalence among children across Asia which was 4.3%. The prevalence was also lower when compared to children in other areas of Japan, especially urban Tokyo [28]. Futamura et al. did a study on Tokyo children in 2005 and the 12-month-period prevalence among those 27,196 schoolchildren with the age range of 6 to 14 years old was 10.9–19.6% [80]. The total prevalence of AD was higher in younger Tokyo schoolchildren as compared to the older ones. Lastly in Japan, a study conducted among Osaka children in 2006 gave a prevalence of 4.6%. A total of 52,213 children in the age range of seven to 12 years old were involved in this study [81]. Figure 5 summarizes AD prevalence data corresponding to the Asian region.

### 3.7. Europe

**Figure 6:**
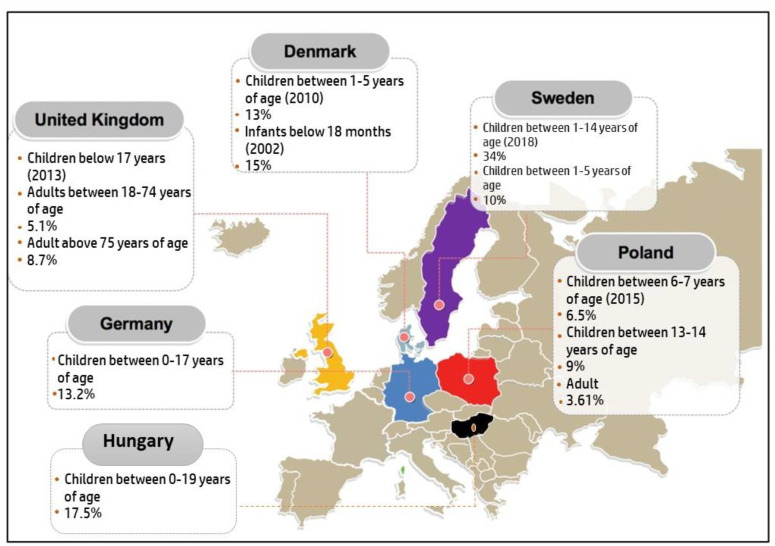
Prevalence of AD in Europe based on the reports published between 2009 and 2019.

A total of six countries published prevalence studies on AD in the recent years which were the UK, Denmark, Sweden Poland, Germany, and Hungary, their results are visualized in Figure 6. In the UK, a study was conducted by Abuabara et al. to measure the prevalence of AD among 8,604,333 UK citizens in the years 1994 and 2013 [3]. The prevalence reported was 12.3% in children 17 years old and younger, 5.1% in adults (18 to 74 years old), and 8.7% in older adults age over 75 years of age. It has been shown that children had the highest prevalence as compared to older age groups. Furthermore, among the over >8 million individuals aged 0 to 99 years, the cumulative lifetime prevalence of atopic eczema was 9.9% and rates of active disease were highest among children and older adults.

In Denmark, it was reported that children at the age of five and born between 1997 and 2011 had a lifetime prevalence of AD of 13%. A total of 972,836 Danish children were involved in this study [55]. Another study involving 52,950 infants under 18 months of age showed a prevalence of 15%. Engebretsen et al. retrieved the participants’ data from the Danish National Birth Cohort (DNBC) and observed the prevalence of AD between 1996 and 2002. Generally, the prevalence of AD among children in Denmark was 14% [82].

In Sweden, the most recent study conducted has observed the prevalence of AD among children between the ages of 1–4 years old. A total of 2215 pre-schoolers were enrolled and showed an AD prevalence of 34% which is the highest seen in this continent based on this literature review. Moreover, the same study conducted by Henriksen et al. in Denmark was also performed among Swedish children [55]. A total of 534,541 children born between 2006 and 2010 were involved in the study to measure the prevalence of AD which was reported as 10%. Furthermore, the majority of children had a debut of AD in infancy. In both, Denmark and Sweden’s boys were more often identified with atopic dermatitis than girls and the risk of atopic dermatitis was high during winter and spring [55,82].

In a paper, published by Schmitz et al. German children between May 2003 and May 2006, were reported to have a 13.2% lifetime prevalence of AD, with a total of 17,450 children in the age range of 0 to 17 years old enrolled in this study [83]. Lastly, a study done in Poland gave the lowest lifetime prevalence compared to other countries on this continent and in all age groups, which were 6.5% in children between 6–7-years-old, 9% in adolescents between 13 to 14 years old, and 3.62% in adults [84].

A study performed in Hungary also showed a growing number of AD cases: the prevalence of AD was investigated in individuals under 19 years of age within the agricultural area of East Hungary. Combined data obtained with the Schultz-Larsen questionnaire on 1158 children were analyzed, and 25% of the index persons were examined by licensed dermatologists. The mean prevalence of AD determined by questionnaires appeared to be 17.5% in the entire study population [85].

### 3.8. Oceania

**Figure 7:**
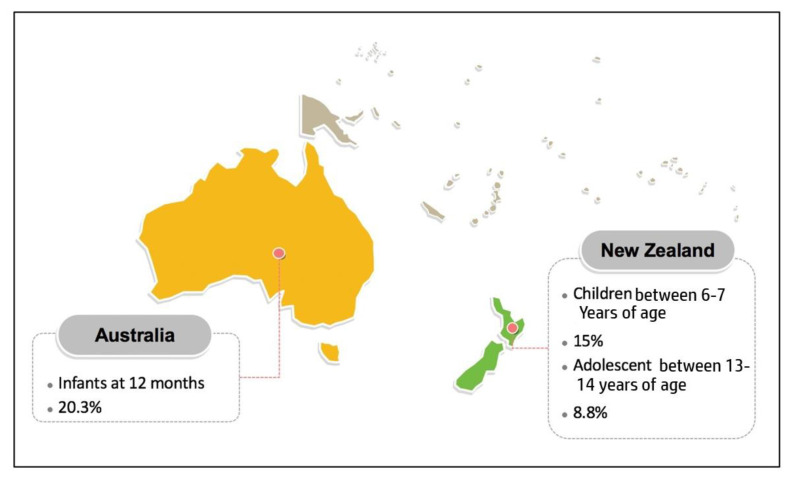
Prevalence of AD in Oceania based on the reports published between 2009 and 2019.

Regarding AD, there are not many prevalence studies available from Australia and New Zealand in the recent years, compared to the other geographical regions. In Australia, it was reported that infants at 12 months had an AD prevalence of 20.3% [86]. This study conducted by Martin et al. aimed to measure AD prevalence between 2008–2011 and involved a total 4972 of infants. Next, Kiwi children between the age of six to seven years and adolescents between the age range of 13 to 14 years old have been associated with an AD prevalence of 15% and 8.8%, respectively [87]. This study is another depiction of children having a higher AD prevalence compared to adolescents (Figure 7).

In short, for data on the prevalence of AD, there were six countries representing Africa, one region (Latin America) and three countries represented America, five countries represented Asia, five countries represented Europe and lastly, two countries represented Oceania during the time period between 2009 and 2019. Next, the prevalence of AD was highest in younger children compared to older children, adolescents, and adults. The highest prevalence was among Sweden children with 34% and the lowest prevalence was reported in Tunisian children with 0.65%.

### 3.9. Incidence of Atopic Dermatitis

There are not many incidence studies available on AD in the recent years [88,89,90]. Most of the studies on incidence rates were published from the European countries which were Norway, Denmark, Sweden, and Germany. In Norway, it was reported that children younger than 6 years of age have had an overall incidence rate of AD increased from 2009 to 2014, with an increment of from 0.028 per person-year to 0.034 per person-year, respectively. The same trend was seen for children younger than 1 years of age [29]. The incidence rate also increased from 0.052 per person-year to 0.073 per person-year respectively. This indicates an increase in the incidence of pediatric AD [29]. A study in Denmark stated that the incidence rate of atopic dermatitis was stable among Danish children corresponding to 1.05 in 1998 to 1.06 in 2011. The same trends were seen in Sweden, as pediatric AD cases in this country had a stable incidence rate of 1.05 in 2007 to 0.99 in 2010 [55]. Lastly in Germany, it was reported that the children between ages 9- to 11- and 16- to 20-year-old have an incidence of 1.7%, and a recurring incidence of 2.4% [91].

## 4. Concluding Remarks

Based on the results on our literature survey, the prevalence of AD was highest in younger children compared to older children, adolescent, and adults. The inconsistencies between results may be attributable to several factors including differences in the age groups defined for study populations, the mechanisms involved in diagnosing AD, and the types of prevalence reported which consisted of point prevalence, period prevalence, and lifetime prevalence. The prevalence stated was point prevalence unless stated otherwise. With regards to the geographic origin of the available studies, there were six countries representing Africa (Tunisia, Rwanda, Namibia, Ghana, Gabon, and Senegal), one region (Latin America) and three countries (United States of America, Brazil, and Canada) representing America, six countries representing Asia (China, South Korea, Malaysia, Japan, Taiwan), five countries representing Europe (United Kingdom, Denmark, Poland Sweden, Hungary, and Germany) and lastly, two countries representing Oceania (Australia and New Zealand). In all the studies across the globe, the highest prevalence was among Sweden children with 34%, and the lowest prevalence was reported in Tunisian children with 0.65%, respectively.

In conclusion, studies included in this narrative review mainly focused on AD and the risk factors associated with this disease. Evidence obtained from included studies shows that AD is a prevalent problem all around the world, both among infants, children, young adults, and adults. Moreover, the prevalence of AD was highest in younger children compared to older children, adolescents, and adults. The significant predictors or most important associated risk factors of AD were age, weather, food, race, ethnicity, place of birth, sex, current working status, and family history of atopy and maternal age. It is necessary to prevent the onset and exacerbation of symptoms, along with improving the quality of life of schoolchildren affected by AD and therefore, appropriate healthcare and environmental authorities should pay special attention to organize the proper health care of these patients and prevention of the manifestation of the disease. There are only a few recent studies on the incidence of AD in the 21st century, and no studies on adults only; most studies have been conducted in Europe and the USA. Lastly, additional epidemiological surveys to investigate allergic disease (AD) and its potential risk factors are needed in the future.

### 4.1. Strength and Limitations

This narrative review included a comprehensive search and a critical assessment of the reviewed studies. The findings reported data from representative population-based epidemiological studies, including those with large representative cohorts, data from several decades, and data from all continents. Therefore, this paper reports on findings in highly diverse settings and populations. Moreover, to identify the relevant studies, the exact definition of AD was followed. However, the definitions of AD may have changed over the decades; for example, the trends in data using doctor-diagnosed AD, self-reported AD using ISAAC criteria, and otherwise-reported AD were quite stable. While similar diagnostic criteria were used in some studies—like the ISAAC or adapted ISAAC criteria—differences in study design and slight differences in the questionnaires used made it difficult to summarize the data. Lastly, some included studies were designed to assess the prevalence and incidence of AD, whereas others reported on AD as a secondary outcome. Many studies lacked data on the participation rate, and data on socioeconomic status of the affected patients. The studies in this review included data from 2009 until 2019.

### 4.2. Comparison with Other Studies

The data present here is also in accordance with data from ISAAC studies, suggesting that there is no clear pattern of prevalence of AD across continents. In a systematic review by Abuabara et al. [92], the prevalence of AD for adolescents/young adults and children was similar to our findings. In addition, a review by Pols et al. reported that the assessed prevalence of AD may vary according to diagnostic methods [93]. More studies are needed using the same validated diagnostic tools and a similar study design.

## Data Availability

Not applicable.
